# Validation of the SkinConnect Nomadic Multi‐Parametric Device for Assessing Stratum Corneum Hydration

**DOI:** 10.1111/jocd.70998

**Published:** 2026-06-23

**Authors:** Frédéric Flament, Thomas Dauxerre, Juliette Rengot, Marie Cherel, Ghislain François, Matthieu Jomier, Guive Balooch

**Affiliations:** ^1^ L'Oréal Research and Innovation Clichy France; ^2^ QIMA Life Sciences, QIMA Newtone SAS Lyon France

**Keywords:** clinical evaluation, connected device, nomadic measurement, skin hydration

## Abstract

**Background:**

Hydration is an essential skin parameter measurable by only a few nomadic devices. Focusing on personalized skincare in retail environments, we designed the SkinConnect, a compact, all‐in‐one device capable of analyzing skin color, microrelief, and hydration. This study constitutes the initial assessment of the SkinConnect, evaluating repeatability, reproducibility, and sensitivity to detect hydration changes, and benchmarking it against the Corneometer.

**Materials and Methods:**

An independent Contract Research Organization recruited 22 females and 22 males and measured skin hydration in triplicate on six body areas (the forehead, a cheek, the chest, both forearms, and a calf). Measurements were taken at one‐hour intervals, including before and after applying a hydrating cream to one forearm. Parallel measurements were conducted using a Corneometer.

**Results:**

Among the 4665 SkinConnect measurements, 0.2% displayed aberrantly high values while 3.1% were null. The mean standard deviation for intra‐device repeatability was 3.68 and inter‐device measurements showed a correlation coefficient of 0.917–0.941. Intra‐device reproducibility showed a correlation coefficient of 0.86–0.88, slightly lower than that of the Corenometer. When benchmarked against the Corneometer, correlation was best described by a logarithmic regression (*r* = 0.73), indicating lower sensitivity to low hydration levels. Nonetheless, the SkinConnect device effectively detected cream‐induced hydration increases and more subtle hydration differences across body areas.

**Conclusion:**

Although further studies are warranted, this work indicates that the SkinConnect enables reliable skin hydration monitoring, making it a compact and efficient solution for multi‐parametric skin assessment and providing access to professional‐grade skin analysis. It may also prove valuable in assessing skin disorders.

## 1 Introduction

Human skin exhibits a remarkable diversity. Ethnicity, genetic background, environment, lifestyle, and age are major contributors to this diversity, accounting for variations in skin type, color, texture, and specific needs. The widespread availability of information via the Internet and the prominent influence of social media have led to unprecedented consumer education in skincare, although this has occasionally resulted in misinformation [1, 2, 3]. Nevertheless, there is a growing awareness of individual skin needs. Consequently, the cosmetics and skincare industry faces consumers who increasingly seek evidence‐based, tailored solutions [4].

This demand for personalized skincare highlights the need for advanced diagnostic tools in retail environments, providing accurate, evidence‐based assessments of individual skin characteristics. Recent advances in skin imaging and Artificial Intelligence (AI) now allow automatic scoring of multiple facial attributes from selfies taken with smartphones, with accuracy comparable to dermatologist evaluations across diverse ethnic groups [5, 6, 7, 8, 9, 10]. Combined with augmented and virtual reality, such tools enhance consumers decision‐making, cosmetic outcomes, and product personalisation [11, 12, 13].

A complementary approach involves portable imaging systems capable of providing detailed surface assessments. The SkinCam is one such device, developed for dermatological and cosmetic applications. It analyses a 25‐mm skin area under controlled multi‐LED illumination without polarization filters, under cross‐ and parallel‐polarization, allowing the evaluation of multiple cutaneous characteristics, including microrelief, scarring, and xerosis [14, 15, 16]. However, the current version lacks the ability to quantify skin hydration.

Hydration is an essential skin marker. It is fundamental for the proper functioning of the skin, particularly in maintaining the homeostasis of the outermost layer, the stratum corneum, which is exposed to a dry external environment. Hydration is vital for maintaining an effective barrier function, ensuring structural integrity, facilitating normal stratum corneum maturation and desquamation, and providing elasticity and resilience [17, 18, 19]. Any imbalance in skin hydration has direct visible consequences, such as the roughness observed in dry skin. Xerosis is also a hallmark of pathological conditions such as atopic dermatitis and psoriasis, which manifest with sensations of tightness and itching alongside reduced quality of life [20]. Conversely, studies on moisturizers have demonstrated a direct relation between increased skin hydration, improved skin smoothness, reduced visibility of fine lines, and enhanced comfort [21]. Hydration also influences the optical properties of the stratum corneum, affecting the skin's reflectivity and transparency [22]. Restoring and maintaining optimal hydration through tailored emollient regimens is therefore not only of biophysical interest but also a cornerstone of xerosis management, suggesting the potential value of objective, routine measurements to guide skincare recommendations and monitor treatment response.

While several approaches are available to measure skin hydration, including direct quantification, the most widely used method is indirect and relies on assessing skin's electrical properties, whether conductance or capacitance [23]. The gold standard in this field is the Corneometer (Courage + Khazaka, Germany), which measures the skin's dielectric properties. These dielectric properties primarily depend on hydration since water has the highest di‐electrical constant among skin's constituents [24, 25]. Sensitive and reliable, the Corneometer provides highly repeatable and reproducible results with limited inter‐device variability [26, 27, 28]. Even though inter‐instrumental comparisons have revealed occasional discrepancies across devices [26]. However, it requires good contact between electrodes and the skin, and various substances can affect measurements if they interfere with the electrical signal [28, 29].

We have employed the conductance approach to create a skin hydration measuring instrument. By combining it with the features of the SkinCam, we have developed a new, extremely compact device—the SkinConnect – designed and optimized for in‐depth skin analysis in a retail environment, while upholding the highest standards of the dermatological and cosmetic industry. Its capabilities to analyze skin images have been previously validated, including the assessment of skin microrelief [14]. This study constitutes the initial validation of SkinConnect's hydration measurement functionality through comparative human measurements versus the reference Corneometer, specifically evaluating: (i) intra‐ and inter‐device repeatability, (ii) reproducibility over time, (iii) benchmarking against the reference standard, and (iv) sensitivity to detect hydration changes induced by moisturizer application and inter‐site variation.

## 2 Materials and Methods

### 2.1 Subjects

This non‐invasive clinical evaluation was conducted independently by a Contract Research Organization (IEC, Lyon, France). It managed all aspects of subject recruitment, measurement acquisition (SkinConnect and Corneometer), data collection, and results quality control.

A total of 44 subjects residing in the region of Lyon (France) were recruited. The cohort comprised 22 males (with low hair density) and 22 females with an age ranging from 19 to 70 years, with the two groups distributed as evenly as possible across this range (mean age ± SD: 49.7 ± 15.8 for the entire group, 51.1 ± 15.5 for the male subgroup, and 48.3 ± 16.4 for the female subgroup).

Participants had no active dermatological conditions, systemic comorbidities known to affect TEWL (e.g., eczema, psoriasis, diabetes, renal or hepatic impairment), or ongoing treatments that could influence skin barrier function (e.g., topical/systemic corticosteroids, retinoids, antihistamines). Subjects also had to adhere to strict pre‐study washout requirements to minimize interference from topical products: no changes in cosmetic routines (study areas) for 2 weeks prior to study; no cosmetics/pharmaceuticals (except usual cleanser) on test sites (forehead, cheeks, chest, forearms, legs) for 72 h prior to study; no products (including water) on test sites on study day; and no caffeine‐containing drinks during testing. To further prevent interference with measurements, female participants were asked to epilate their calves 7 days prior to the study or to shave them the day before the study. Similarly, male participants were instructed to shave or epilate their cheeks, chest, and calves 7 days before the study and the day before.

### 2.2 The SkinConnect Device

**FIGURE 1:**
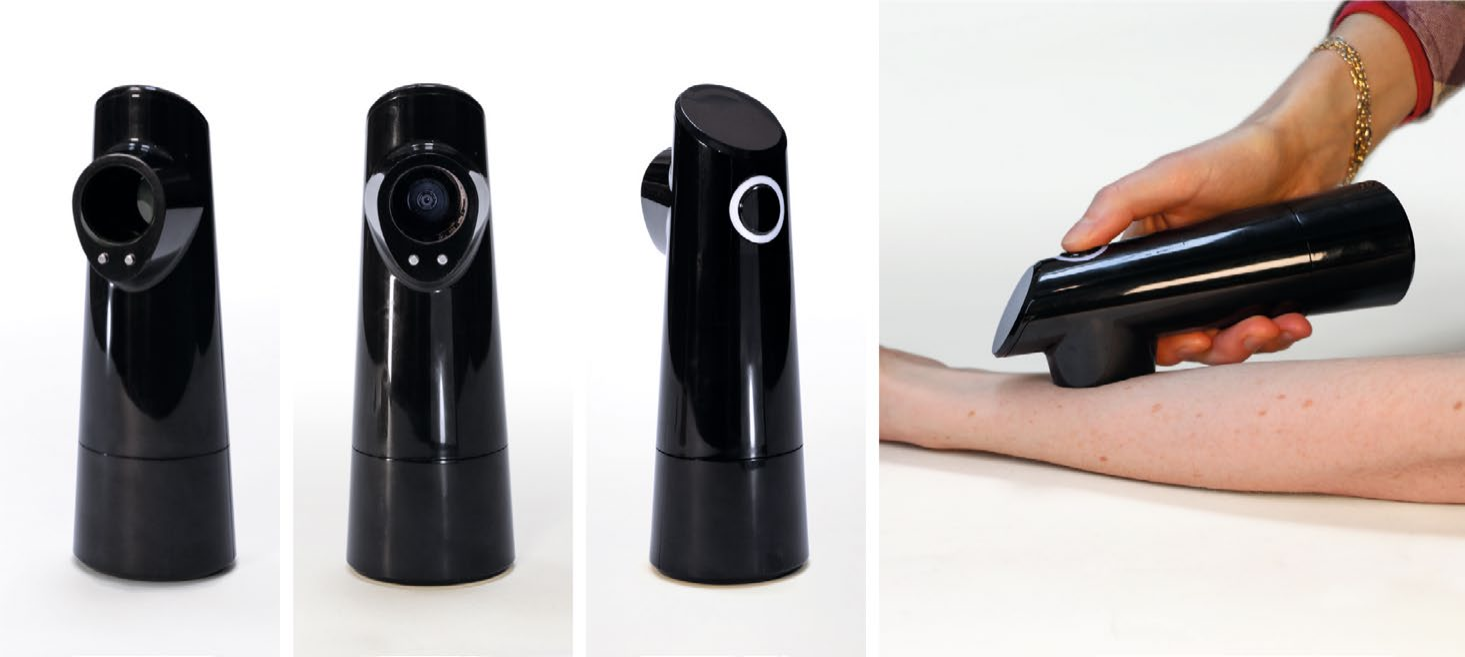
The SkinConnect device.

The SkinConnect is an enhanced version of the previously described SkinCam device [14], now incorporating a skin hydration measurement functionality (Figure 1). This compact, nomadic device generates its own Wi‐Fi network, allowing control via a smartphone, tablet, or computer web‐based application. Equipped with a high‐resolution CMOS sensor, the SkinConnect analyses skin colourimetric parameters by capturing images of a 25 mm diameter‐large skin area illuminated by 12 evenly spaced white LEDs. Within seconds, images are captured without polarization or under cross‐ and parallel polarization, as well as using UV illumination. Furthermore, by sequentially illuminating the LEDs and acquiring parallel‐polarized images, the device enables the analysis of the skin microrelief.

Skin hydration measurement is achieved by quantifying the conductance of the skin subjected to a weak, 3.3 kHz electric current between the two electrodes. These electrodes, separated by 10 mm, are localized at the extremity of the cache covering the CMOS sensor and the LEDs, cache that comes in contact with the skin when performing acquisitions. As the SkinConnect provides raw skin hydration readings on a 0–38 scale, which is not easily interpretable, all measurements were normalized to a 0–100 scale before analysis by dividing reading by 38 and multiplying the result by 100.

For every acquisition, the SkinConnect performs both image captures and a skin hydration measurement.

### 2.3 Evaluation of the SkinConnect Devices

**FIGURE 2:**
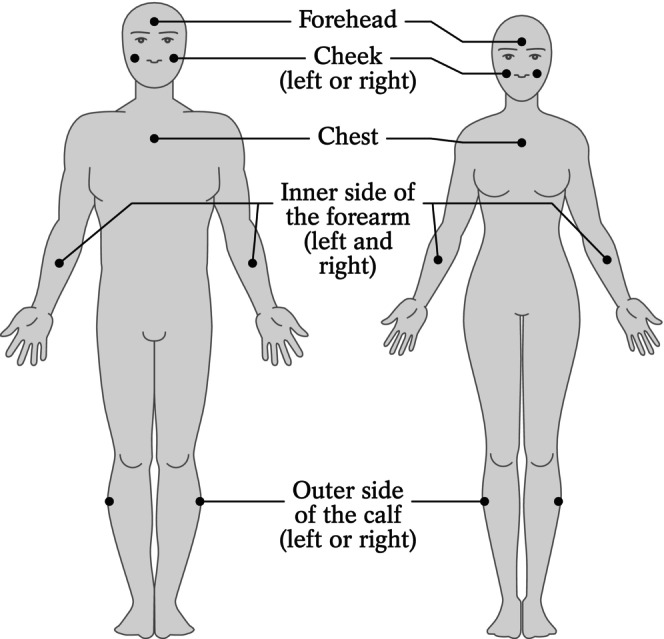
Body regions where measurements were performed using the SkinConnect devices and the Corneometer.

At the study outset, 4 × 4 cm square zones were delineated on the subjects' forehead, left or right cheeks, chest, left and right forearms, and left or right calves (Figure 2). Following a 20‐min acclimatization period under standardized conditions (20°C–23°C, 50% ± 10% relative humidity), triplicate measurements with repositioning were taken on the six zones. Additional triplicate measurements of each zone were performed 1 h later. All triplicate measurements of the six zones at both time points were conducted with three different SkinConnect devices. All acquisitions with the SkinConnect were performed by a single technician.

To compare the results of the SkinConnect devices with those from a reference device, triplicate measurements of each zone at both time points were also acquired using a Corneometer CM 825 (Courage + Khazaka Electronic, Germany). Acquisitions with the Corneometer were performed by the same technician conducting SkinConnect acquisitions.

Intra‐device repeatability was assessed by comparing the triplicate measurements obtained using the three SkinConnect devices at both time points (T0 and T1 hour) with those of the Corneometer. Intra‐device reproducibility was evaluated by comparing the mean value of the triplicate measurements performed at T0 and T1 hour with the three SkinConnect devices with those from the Corneometer. Inter‐device repeatability was determined by comparing the mean values of the triplicate measurements obtained at the same time point across the three SkinConnect devices.

### 2.4 Monitoring of the Effect of a Hydrating Cream

To investigate the ability of the SkinConnect device to detect changes in skin hydration, a hydrating cream (2 mg/cm^2^) was applied topically to a randomly selected forearm zone immediately after the initial triplicate measurements. Cream applications were performed by different trained technicians using a standardized procedure; the treated forearm was blinded to the measurement technician. The effects of the cream were assessed by performing triplicate measurements at baseline—before cream application—and 1 h later, after the cream had been removed by three wipes. Results were compared to those obtained from an untreated, equivalent skin area on the opposite forearm. Results were also compared with those obtained using the Corneometer, which involved triplicate measurements of both forearm zones at both time points.

The cream used for this experiment is a widely available commercial oil‐in‐water emulsion (Lait Corporel L'Original, Biotherm, France). The primary ingredients acting on skin hydration are olive oil, glycerin, and urea. Its complete INCI formula is aqua/water, *Olea europaea* fruit oil, glycerin, dimethicone, propylene glycol, triethanolamine, limonene, isopropyl palmitate, *Citrus aurantium dulcis* oil, stearic acid, paraffinum liquidum, cetyl alcohol, *Glycine soja* oil, *Citrus grandis* peel oil, urea, aspartic acid, paraffin, carbomer, glucose, fructose, glyceryl stearate, dimethiconol, sodium lauroyl oat amino acids, myristic acid, palmitic acid, alanine, sucrose, *Vitreoscilla* ferment, xanthan gum, glutamic acid, dextrin, ethylhexylglycerin, hexyl nicotinate, hexylene glycol, tocopherol, sodium dehydroacetate, phenoxyethanol, linalool, citral, fragrance.

### 2.5 Statistical Analysis

The following statistical analyses were applied exclusively to the two experimental series described below: the effect of the hydrating cream and the comparison of hydration levels across body regions. Results are presented as the mean ± standard deviation (SD). Data distribution was evaluated using the Shapiro–Wilk test (α < 0.05). Normally distributed data were compared using ANOVA followed by Tukey's HSD *post hoc* tests. For non‐normally distributed data, the Kruskal‐Wallis test was employed, followed by pairwise comparisons using Dunn's tests. In the case of Tukey's HSD and Dunn's tests, *p*‐values were adjusted using the Bonferroni correction: six pairwise comparisons were performed for the hydrating cream analysis (adjusted threshold: *p* < 0.0083), and ten pairwise comparisons for the body‐region analysis (adjusted threshold: *p* < 0.005). Throughout the text, all reported *p*‐values are Bonferroni‐corrected, with *p* < 0.05 retained as the threshold for statistical significance.

## 3 Results

### 3.1 Measurements Overview

The analysis included data from 44 subjects of both sexes, with triplicate measurements performed on six different body locations (the forehead, one cheek, the chest, both forearms, and a calf) and at two time points (T0 and 10 h later). This resulted in a total of 1584 skin hydration measurements collected using the Corneometer. With the three SkinConnect devices tested, the total number of measurements should have been 4752. However, due to handling problems or data loss during Wi‐Fi transfer, 87 acquisitions (1.8%) were lost, leaving 4665 measurements.

**FIGURE 3:**
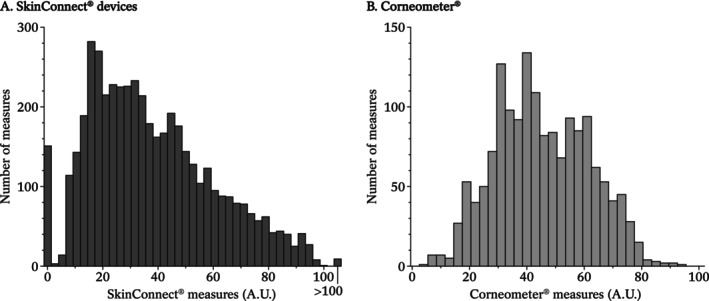
Histograms of the measurements on all skin zones and time points (a) for all three SkinConnect devices and (b) the Corneometer. Histograms present measurements, which are grouped by three for the Corneometer to achieve a similar presentation.

To provide an initial assessment of the performance of the three SkinConnect devices, we plotted the frequency of each measurement (Figure 3A). The majority of readings (4515, 96.8%) fall within the 1 to 100 value range. Only seven measurements (0.2%) exceed a value of 105, representing aberrant measurements. However, caution is warranted when considering these seven values. Six originate from baseline measurements of forehead skin hydration using two different SkinConnect devices on a single subject. Furthermore, the third SkinConnect device recorded notably high hydration levels (76, 66, and 66), as did the Corneometer (95, 90, and 92). Apart from these high readings, 143 measurements (3.1%) resulted in a value of 0, indicating that no measurement was performed. As a result, subsequent analyses focus on valid measurements, excluding null and aberrant values.

When performing a similar analysis with the Corneometer measurements (Figure 3B), no null or aberrant values are observed. All readings range between 5 and 95.

### 3.2 Intra‐Device Repeatability

We then assessed the intra‐device repeatability by analyzing the variability of triplicate measurements obtained by the same device on the same body at an identical time point. Metrics included the standard deviation of triplicates, the coefficient of variation, and the maximum difference across triplicates (Table 1).

**TABLE 1:** Intra‐device repeatability of triplicate measurements.

	SkinConnect 1	SkinConnect 2	SkinConnect 3	All SkinConnects	Corneometer
Standard deviation					
Mean	3.32	4.05	3.65	3.68	2.41
Median	2.63	3.04	3.04	2.63	2.08
Minimum	0.00	0.00	0.00	0.00	0.00
Maximum	18.67	33.53	24.45	33.53	12.58
Coefficient of variation					
Mean	0.09	0.13	0.12	0.12	0.06
Median	0.08	0.09	0.09	0.09	0.04
Minimum	0.00	0.00	0.00	0.00	0.00
Maximum	0.43	0.83	0.69	0.83	0.39
Maximum differences					
Mean	6.19	7.61	6.82	6.89	4.64
Median	5.26	5.26	5.26	5.26	4.00
Minimum	0.00	0.00	0.00	0.00	0.00
Maximum	36.64	60.53	47.37	60.53	25.00

The mean standard deviation for triplicate measurements across all three SkinConnect devices is 3.68, with values ranging from 3.32 to 4.05, depending on the device. This value is slightly higher than that of the Corneometer, which exhibits a mean standard deviation of 2.41. While the median of the standard deviation is relatively similar across all devices, including the Corneometer, the SkinConnect devices tend to show some higher standard deviations, especially device #2.

Similar trends are obtained when analyzing the coefficient of variation (the ratio between the standard deviation and the mean value of triplicate measurements) and the maximum difference between triplicate measurements. These suggest that while the SkinConnect device generally provides repeatable measurements, it can occasionally result in inconsistent measurements, especially with device #2.

### 3.3 Inter‐Device Repeatability

To further explore the variability of measurements, we analyzed the variability of the means of triplicate measurements performed at an identical body site and timepoint by three different SkinConnect devices. Parameters evaluated were the Pearson correlation coefficients between device pairs, the standard deviation of means, the coefficient of variation, and the maximum differences across devices.

The correlation coefficients between the triplicate values of the three SkinConnect devices are high (Table 2), ranging from a minimum of 0.917 between devices #2 and #3 to a maximum of 0.941 between devices #1 and #3. In more detail, the mean standard deviation of the triplicate measurements is 4.06, with a low mean coefficient of variation of 0.13 and an average maximum difference of 7.68 (Table 3). Out of 448 triplicate measurements across the three devices, no differences were found in only three cases. Besides, the median of the standard deviation, coefficient of variation, and maximal difference is lower than their respective means, indicating that the inter‐device variability was generally low. However, the highest maximum difference is 65.79, though only 23 instances (5.1%) exceed a difference of 20.

**TABLE 2:** Coefficients of correlation between the triplicate measurements obtained with the three SkinConnect devices.

	SkinConnect 1	SkinConnect 2	SkinConnect 3
SkinConnect 1	—	0.938	0.941
SkinConnect 2	0.938	—	0.917
SkinConnect 3	0.941	0.917	—

**TABLE 3:** Inter‐SkinConnect device repeatability of triplicate measurements.

	Standard deviation	Coefficient of variation	Maximal difference
Mean	4.06	0.13	7.68
Median	3.08	0.09	6.14
Minimum	0.00	0.00	0.00
Maximum	36.34	1.02	65.79

### 3.4 Intra‐Device Reproducibility

Another important point is reproducibility, the ability of the devices to provide consistent results over time. We assessed this point for each device, analyzing the Pearson correlation coefficient between the mean values of triplicate measurements at baseline and 1 h later. For this analysis, measurements from forearms treated with the hydrating cream were excluded.

**FIGURE 4:**
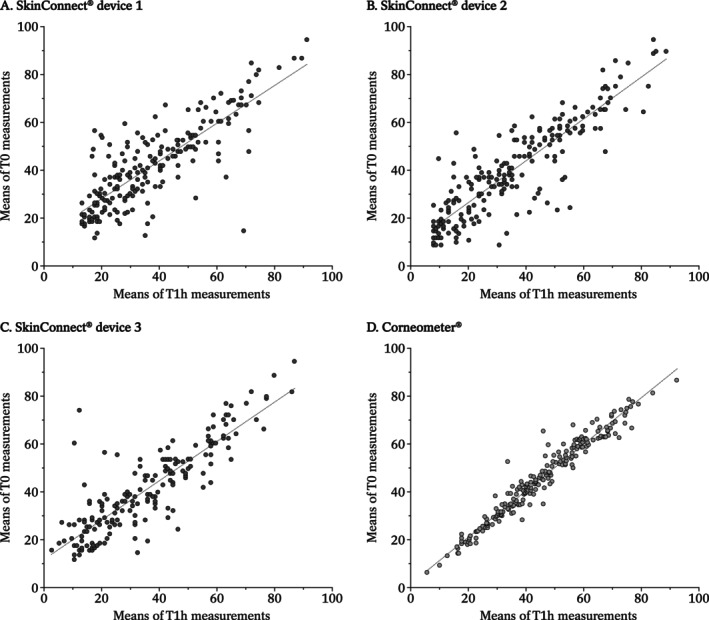
Correlation between the means of triplicate measurements at baseline and 1 h later for the three SkinConnect devices and the Corneometer.

The Corneometer demonstrated excellent reproducibility, with data points closely aligned along the linear regression line (Figure 4D) and a coefficient of correlation of 0.98 (*p* < 0.001). When performing an identical analysis with the three SkinConnect devices, points are more scattered (Figure 4A–C), occasionally exhibiting poor reproducibility. Nonetheless, the overall correlation coefficient for the three SkinConnect devices is high—0.87 (*p* < 0.001) – with individual values ranging from 0.86 for device #1 and 3 to a maximum of 0.88 for device #2. Yet, interquartile range analysis shows that 6% of reproducibility data (37 out of 575) should be considered as outliers. When excluding these outliers, the coefficient of correlation between measurements performed at the two time points reached 0.93 for the three SkinConnect devices (*p* < 0.001), with a minimum of 0.92 for device #1 and a maximum of 0.94 for device #2.

### 3.5 Benchmarking of SkinConnect Devices Against the Corneometer

To complete the assessment, we analyzed the correlation between the means of measurements from the three SkinConnect devices and those from the Corneometer. The objective of this comparison was to benchmark the SkinConnect against the Corneometer as the gold standard in skin hydration measurement, rather than to establish clinical equivalence between the two devices, which differ substantially in their intended use context and measurement principle.

**FIGURE 5:**
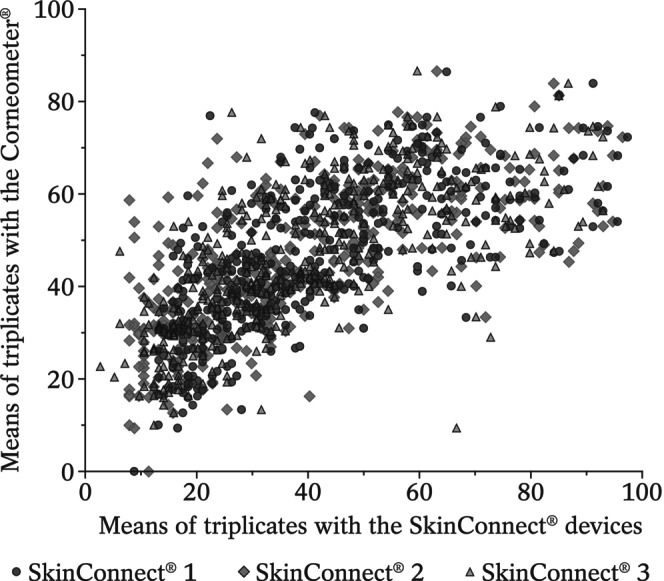
Correlation between the means of triplicate measurements from the three SkinConnect devices and the Corneometer.

The Pearson coefficient of correlation calculated from linear regression between results from the SkinConnect devices and those of the Corneometer is 0.71 (*p* < 0.001) whatever the device (Table 4). However, graphical analysis (Figure 5) revealed that a simple linear regression is not the most appropriate model due to an inflection at lower values. Logarithmic regression yielded better correlations, suggesting that SkinConnect devices exhibit lower sensitivity than the Corneometer at lower hydration levels.

**TABLE 4:** Correlation between the means of triplicate measurements with the SkinConnect devices and the means of triplicates from the corneometer.

	SkinConnect 1 vs. corneometer	SkinConnect 2 vs. corneometer	SkinConnect 3 vs. corneometer
Linear regression	0.71	0.71	0.71
Logarithmic regression	0.74	0.72	0.73

### 3.6 Evaluation of the Effect of a Hydrating Cream

**FIGURE 6:**
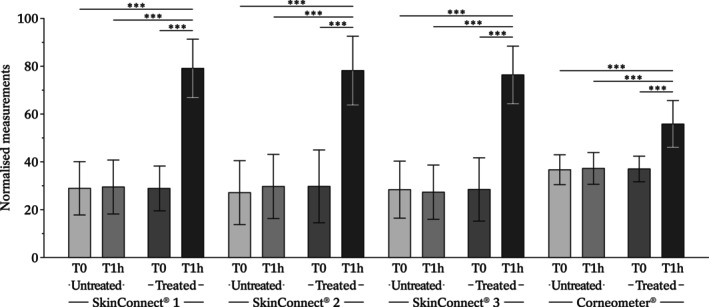
Skin hydration measured by the three SkinConnect devices and the Corneometer in an untreated forearm zone and the opposite forearm area that received topical application of a hydrating cream. Measurements are performed at T0 (before cream application) and 1 h later. For the statistical significance: ***: *p* < 0.001.

Besides providing repeatable and reproducible measurements, skin hydration measuring devices must be sensitive enough to capture any change in skin hydration. Thus, we monitored the change in skin hydration induced by the topical application of a hydrating cream (Figure 6).

In cream‐treated forearm areas, 1 h after application, the three SkinConnect devices detect a significant increase in skin hydration (163%–174% depending on the device; *p* < 0.001). This increase is significant not only relative to the treated forearm area prior to cream application but also compared to the opposite untreated forearm zone at both baseline and 1 h later (*p* < 0.001 for all comparisons). Besides, no significant differences are observed between both forearms at baseline and in the skin zone of the untreated forearm 1 h later.

Similar trends are obtained with the Corneometer, though the cream‐induced increase in hydration is less pronounced (51%).

### 3.7 Assessment of Skin Hydration in Different Body Regions

**FIGURE 7:**
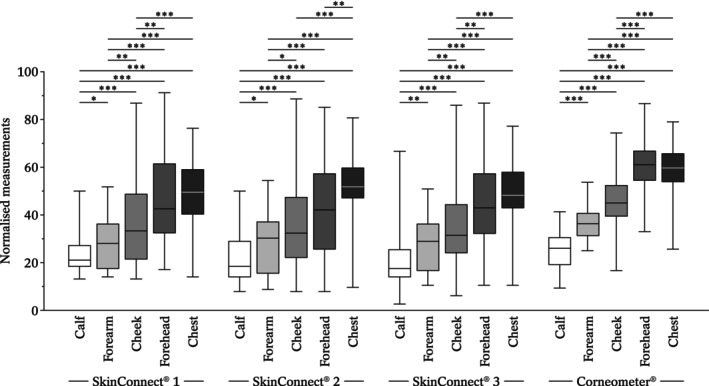
Skin hydration measured by the three SkinConnect devices and the Corneometer in different body regions. For the statistical significance: *: *p* < 0.05, **: *p* < 0.01, and ***: *p* < 0.001.

Finally, the ability of the SkinConnect devices to differentiate hydration levels across various body regions was evaluated. Thus, we compared measurements from the different skin zones assessed at both time points, excluding data from the cream‐treated areas (Figure 7).

The Corneometer measurements indicate no hydration difference between the forehead and the chest but reveal significant differences in all other areas. The lowest hydration level is found on calves, followed by the forearm, and the cheek; maximum and similar levels being found on the forehead and chest. SkinConnect devices #1 and #3 yielded similar results, albeit with greater variability, especially at the level of the forehead. Only the SkinConnect device #2 was slightly less precise, failing to detect a significant difference between the cheek and the forehead, even though the lack of significance is at the very limit (*p* = 0.053). It is also the only device indicating a difference between the forehead and the chest.

## 4 Discussion

Measurements of skin hydration using three different SkinConnect devices demonstrated their intra‐ and inter‐device repeatability, as well as their intra‐device reproducibility. Importantly, the SkinConnect devices effectively detected the increase in skin hydration induced by a hydrating cream, as well as the more subtle hydration differences existing between various body locations, differences that have been previously documented [30, 31, 32].

When benchmarked against the Corneometer, widely regarded as the reference for skin hydration measurement, SkinConnect results exhibit a correlation coefficient of 0.72–0.74. While this correlation is lower than that of high‐end, dedicated research and evaluation devices such as the Skicon 200, Nova DPM, and DermaLab [33], it is comparable to that of the SkinUp (0.70–0.80) [34], which is designed for nomadic measurements, and significantly higher than that of the GPSkin Barrier (0.21–0.44) [35]. Furthermore, the correlation coefficients for triplicate measurements across different SkinConnect devices are high (0.917–0.941), indicating good inter‐device repeatability. These values are similar to those reported for the CM 825 Corneometer (0.89–0.94) [36].

However, seven of the SkinConnect readings exceed a value of 105. Six of these are baseline triplicates of the forehead from a single subject who, according to the third SkinConnect device and the Corneometer, presented very high hydration levels. This suggests that the subject may have had on the forehead a substance interfering with measurements, leaving only a single unexplained aberrant value, just 0.02% of the total readings. Additionally, 3.1% of the acquisitions resulted in a value of 0, indicating a measurement failure. Since these missing results occurred sporadically across all three devices, an electronic defect is unlikely. Furthermore, the acquisitions and transfer of valid skin images indicate that Wi‐Fi transmission failure can be excluded. Consequently, the absence of measurements is most likely attributable to the absence of contact between the electrodes and the skin. Since all subjects were shaved in the areas where measurements were conducted, the known interference of hair with the electrical‐based skin hydration measurements cannot be invoked [37]. Improper positioning of the SkinConnect device therefore appears to be the most plausible cause. Indeed, the skin hydration measuring electrodes protrude from the extremity of the relatively large CMOS sensor/illuminating LEDs cache. This cache, with an external diameter of 33 mm, is much larger than the skin hydration probes of all other instruments. Therefore, particular attention is required to ensure proper contact during acquisition, especially on curved body areas. Since reducing the size of the cache is hardly feasible, emphasizing the importance of correct positioning of the SkinConnect device will be essential.

Further characterization of the SkinConnect device would be valuable. This initial study was conducted exclusively in a Caucasian cohort from a single region (Lyon, France), which may limit generalisability. It will be essential to assess the reliability of the device across various phototypes and ethnic groups, given known differences in baseline hydration levels between populations. Nevertheless, Caucasian facial skin typically exhibits relatively low average hydration [32], suggesting potential suitability for detecting changes, but this assumption requires validation in more diverse cohorts and climates to confirm performance consistency. Further exploration of SkinConnect's hydration measurement characteristics is also warranted. The correlation with the Corneometer, which is known for its excellent sensitivity to low hydration levels [33], indicates that the SkinConnect device does not possess the same sensitivity level. It remains to be determined under which circumstances this limitation might prevent the device from discriminating differences in dry skin conditions, especially as dry and rough skin can compromise measurement accuracy by impeding the necessary close contact between the skin and electrodes [28].

Another important parameter is the depth at which skin hydration is measured. The depth of Corneometer measurements is claimed to be 10–20 μm to assess only superficial stratum corneum hydration [38], even though a study suggests this depth is closer to 45 μm [39], thereby including underlying skin layers. The measurement depth of the SkinConnect device is therefore an important consideration. Additionally, the sensitivity of SkinConnect's hydration measurements to various chemicals warrants further examination, as electrical‐based skin hydration measurements, whether capacitance‐ or conductance‐based, do not exclusively quantify water content but also respond to endogenous and topically applied polar compounds [28]. Nevertheless, the impact of such interferences should have been limited in the current study due to the strict cosmetic routine and wash‐out period participants were asked to follow.

Independently of the intrinsic qualities and limitations of the SkinConnect, it is important to recognize the tight interconnection existing between skin hydration and transepidermal water loss (TEWL). The stratum corneum hydration depends on both water influx from the dermis and evaporative loss through the barrier [40]. Nevertheless, TEWL varies substantially across anatomic sites, ranging from 4 to 10 g/m^2^/h at the level of the calf to 15–25 g/m^2^/h at the level of the forehead [41, 42], also significantly varying across individuals (20%–40%) [43]. Consequently, comparing hydration values across sites without accounting for underlying TEWL differences can exaggerate or underestimate inter‐site differences which we did not do as our goal was solely to show that the SkinConnect could detect subtle variations existing across body sites. While TEWL co‐measurement is essential for a comprehensive interpretation of hydration, this study focused solely on demonstrating SkinConnect's ability to detect physiologic hydration gradients across sites, as successfully achieved.

Despite these considerations, the SkinConnect device represents a promising all‐in‐one, compact solution enabling colourimetric, microrelief, and hydration analysis of the skin. Primarily designed for retail environments but based on state‐of‐the‐art dermatological and cosmetic industry standards, it will make these standards accessible to consumers. However, it is unlikely that this device can be effectively used by consumers themselves. Hydration measurements require proper electrode‐skin contact and this proper contact of the electrode‐bearing SkinConnect's cache is also indispensable for high‐quality image capture. The work of Grinich et al. [44] on the use of the GPSkin Barrier by untrained and trained subjects highlights the importance of education in achieving reliable test–retest consistency. Consequently, self‐use by consumers appears improbable, and it will be essential to ensure that SkinConnect device measurements are performed by trained personnel and that their expertise is maintained over time.

## 5 Conclusions

The SkinConnect device offers a compact and efficient solution for rapid, multi‐parametric skin analysis, including the measurement of skin hydration. While there remains room for further improvement, it can make professional‐grade skin assessment widely accessible, thereby contributing to the development of evidence‐based, personalized skincare solutions. Furthermore, its capability to evaluate multiple skin parameters, many of which are markers of dermatological conditions, could support the monitoring of skin diseases such as atopic dermatitis, psoriasis, etc. As such, the SkinConnect device should also be of significant value to both dermatologists and patients.

## Author Contributions

Conceptualization, F.F; methodology, T.D; validation, J.R, M.C and G.F.; formal analysis, T.D, J.R, M.C, G.B, M.J.; investigation F.F.; resources, F.F; data curation, G.F, M.J, G.B; writing – original draft preparation, F.F; writing – review and editing, F.F, G.B; visualization, F.F; supervision, F.F; project administration, F.F; funding acquisition, F.F. All authors have read and agreed to the published version of the manuscript.

## Funding

This study was funded by L'Oréal S.A., Research & Innovation.

## Ethics Statement

The study was conducted in accordance with the guidelines of the Declaration of Helsinki of 1964 and its later amendments and was conducted with OECD Good clinical Practices (GCP). The study was approved for Clinical Research (No. 2025‐A02827‐42).

## Consent

Written informed consent to participate in the study and for the publication was provided by all patients. We thank the participants in the study for their involvement.

## Conflicts of Interest

F.F., T.D., and G.B. are full‐time employees of L'Oréal. J.R., M.C., G.F., and M.J. are full‐time employees of Qima Newtone.

## Data Availability

The data that support the findings of this study are available from the corresponding author upon reasonable request.
